# A Biological Study of Composites Based on the Blends of Nanohydroxyapatite, Silk Fibroin and Chitosan

**DOI:** 10.3390/ma15155444

**Published:** 2022-08-08

**Authors:** Anna Tuwalska, Alina Sionkowska, Amadeusz Bryła, Grzegorz Tylko, Anna Maria Osyczka, Michele Laus, Lucy Vojtová

**Affiliations:** 1 Department of Biomaterials and Cosmetic Chemistry, Faculty of Chemistry, Nicolaus Copernicus University in Toruń, 87-100 Toruń, Poland; 2 Institute of Environmental Science, Faculty of Biology, Jagiellonian University, 30-387 Kraków, Poland; 3 Department of Biology and Cell Imaging, Faculty of Biology, Institute of Zoology and Biomedical Research, Jagiellonian University, 30-387 Kraków, Poland; 4 Department of Science and Technological Innovation, University of Eastern Piedmont “A. Avogadro”, 15121 Alessandria, Italy; 5 CEITEC—Central European Institute of Technology, Brno University of Technology, 612 00 Brno, Czech Republic

**Keywords:** silk fibroin, chitosan, hydroxyapatite, biological study, mesenchymal stem cells, bone regeneration, osteogenesis

## Abstract

In this work, the biological properties of three-dimensional scaffolds based on a blend of nanohydroxyapatite (nHA), silk fibroin (SF), and chitosan (CTS), were prepared using a lyophilization technique with various weight ratios: 10:45:45, 15:15:70, 15:70:15, 20:40:40, 40:30:30, and 70:15:15 nHA:SF:CTS, respectively. The basic 3D scaffolds were obtained from 5% (*w*/*w*) chitosan and 5% silk fibroin solutions and then nHA was added. The morphology and physicochemical properties of scaffolds were studied and compared. A biological test was performed to study the growth and osteogenic differentiation of human bone marrow mesenchymal stem cells (hMSCs). It was found that the addition of chitosan increases the resistance properties and extends the degradation time of materials. In vitro studies with human mesenchymal stem cells found a high degree of biotolerance for the materials produced, especially for the 20:40:40 and 15:70:15 (nHa:SF:CTS) ratios. The presence of silk fibroin and the elongated shape of the pores positively influenced the differentiation of cells into osteogenic cells. By taking advantage of the differentiation/proliferation cues offered by individual components, the composites based on the nanohydroxyapatite, silk fibroin, and chitosan scaffold may be suitable for bone tissue engineering, and possibly offer an alternative to the widespread use of collagen materials.

## 1. Introduction

Materials engineering creates many possibilities regarding overcoming civilization diseases and helps to prolong human life accompanied by health and comfort. The bony skeleton is a remarkable organ that serves both a structural function, providing mobility, support, and protection for the body, and also a reservoir function, as the storehouse for essential minerals for the proper function of the whole organism [1]. Therefore, it is important to strive for the reconstruction of natural bone, restoring its functions, and not only its endurance but also biological and cellular activity. In the treatment of bone defects, scaffolds fabricated from biodegradable materials can provide a crawling bridge for new bone tissue in the gap and a platform for cells and growth factors to play a physiological role, which will eventually be degraded and absorbed into the body before being replaced by new bone tissue [2]. Hasany M. et al. observe that, nowadays, different types of decomposable polymers have been used in the fabrication of porous scaffolds. Remarkably, much focus has been given to polymers derived from natural sources owing to their chemical versatility and extracellular matrix that support excellent cellular interactions [3,4]. Natural bone is composed of hydroxyapatite (65–70%), collagen (18–25%), and water (10–12%) [5]. In several laboratories, the work to develop new materials based on the blending of two or more polymers and inorganic nanoparticles is intensive. New materials based on the blending of two polymers or biopolymers which contain nanoparticles can be used as an implant in the context of both hard and soft tissues. Nanostructured materials can be achieved through the intercalation of inorganic nanoparticles in a polymeric matrix through the addition of the particles or by using the precipitation technique to obtain the nanoparticles in situ. However, as Almer J.D. et al. indicated, it is extremely difficult to connect nanoparticles with natural polymers to obtain a homogenous composite [6]. Nevertheless, according to Aksekili A.M.E. et al. [7], scaffolds with different types of composition with hydroxyapatite (HA) appear promising for the treatment of pseudoarthrosis, due to their biocompatibility and long biodegradation time. Furthermore, in the review by Miriam Filippi et al. [8], we find many examples of different materials based on natural polymers and hydroxyapatite, such as hyaluronic acid, keratin, fibrin, gelatin, silk fibroin (SF), collagen, heparin, chitosan (CTS), alginate, starch, agar, dextran, cellulose, carrageenans or gellan gum. Each of these substances and their combinations with each other provide new opportunities to obtain the appropriate desired properties for bone reconstruction using three-dimensional material. In particular, the optimum properties for such biomaterials may vary considerably, depending on the location, the size of the bone defect, and the type of bone lost (cortical or cancellous).

In our previous article [9], we highlighted how many possible forms are offered in chitosan and silk fibroin composites. Silk is a very interesting natural polymer, which is attracting scientists’ attention especially due to the extraordinary strength properties of silk threads (which are stronger than the strongest synthetic fiber, Kevlar 49, used in bulletproof vests) [10]. Kadumudi, Firoz Babu, et al. stated that silk fibroin is characterized by crystalline β-sheets, α-helices, and amorphous random coils. The β-sheets are particularly important because they underlie silk’s strength and stability [11]. However, three-dimensional silk fibroin scaffolds, prepared by the lyophilization technique method, although they are appropriately porous, are also very brittle and unstable. Despite their high degree of biocompatibility, the resistance of scaffolds fabricated from silk fibroin must be increased. On the other hand, Dinoro J., et al. indicated the simplicity of modifying polysaccharides’ properties (including chitosan) and their cationic nature, which is important for bone tissue engineering applications, as chitosan can form polyelectrolyte complexes [12]. Chitosan is a linear polysaccharide composed of glucosamine and N-acetyl glucosamine units linked by β (1-4) glycosidic bonds. There are many different forms of pure chitosan, which differ according to their degree of deacetylation (DD) and molecular weight [13]. The flexible design options for chitosan-based materials and their possible combinations with silk fibroin (thanks to its anionic nature) inspired this research.

There are few reports in the literature on the properties of usia ng 5% (*w*/*w*) chitosan (80% DD, low molecular weight) solution, and our recent studies have shown that materials made with this concentration with the addition of hydroxyapatite and silk fibroin, possess similar mechanical properties to natural bone [8]. Continuing our research, we investigated the properties of three-dimensional materials based on nanohydroxyapatite, silk fibroin, and chitosan (nHA/SF/CTS) in new proportions that have not been found in the literature so far.

The aim of our study was to investigate the effect of the addition of nanohydroxyapatite on the cellular response and mechanical properties of composites with varying proportions of their components. Attention was also paid to the shape of the pores of the materials depending on the various proportions of their components and the correlation between the increased cellular response and the elongated-spindle shape of the pores. Material degradation tests were also performed which, depending on the proportions of the individual scaffold components used, were characterized by differing degrees of disintegration. The results may be useful for designing the composition of the implant depending on the size of the bone loss to be replaced. Moreover, the properties of the nHA:SF:CTS materials obtained were compared to the collagen-based material and nanohydroxyapatite, which is the most frequently chosen material for bone reconstruction.

## 2. Materials and Methods

Type I collagen (Coll) from bovine skin was purchased from Collado, Brno, Czech Republic. Silk (SF) was obtained from *Bombyx mori* cocoons. *Bombyx mori* cocoons were kindly supplied by the President of “Polish Silk Ltd.”, Milanówek, Poland.

Chitosan (CTS) with low molecular weight was supplied by the Sigma–Aldrich Company (Poznan, Poland). Chitosan properties such as deacetylation degree (80%) and molecular weight (1.9 × 10^5^) were determined using methods described in the literature [14,15].

Hydroxyapatite was supplied by the Sigma-Aldrich Company (Poznań, Poland). A solid powder form with a particle size (<200 nm) was intentionally used. It was examined that nanostructured hydroxyapatite (nHA) is expected to have better bioactivity than coarser crystals [16].

Na_2_CO_3_, NaOH, Methanol was supplied by the Chempur Company, Piekary Śląskie, Poland.

1-(3-dimetylaminopropyl)-3-ethylcarbodiimide hydrochloride (EDS) and N-hydroxysuccinimide (NHS) was supplied by the Sigma–Aldrich Company (Poznan, Poland).

### 2.1. Fabrication of the Scaffolds

Chitosan, silk fibroin, or collagen (type I) solution was combined in appropriate concentrations and proportions with the addition of nanohydroxyapatite (as in the previously described procedure [17]).

The cocoons were boiled for 1 h in an aqueous solution of 0.5% Na_2_CO_3_ according to the procedure described in the literature [18] by the method used previously by Ajisawa [19]. Then the fibroin solution was filtered and dialyzed against distilled water for 3 days to yield a fibroin aqueous solution. The final fibroin concentration was 5% (which was determined by weighing the remaining solid after drying).

Chitosan solution was prepared by dissolving 5 wt% chitosan in 0.5M acetic acid, with a mechanical stirrer, then heating it to 40 °C until the ingredients dissolved (over 48 h, with a break overnight). An equal volume of the same concentration of silk fibroin was added. Similarly, nanopowder of hydroxyapatite was added to prepare a mixture of nHA:SF:CTS with ratios (*w*/*w*) of 10:45:45, 15:15:70, 15:70:15, 20:40:40, 40:30:30, and 70:15:15, respectively. The three-component mixture was homogenized using a magnetic stirrer (4 h) and ultrasound treatment (15 min). The mixed solution was poured into 24-well polystyrene culture plates, and frozen overnight in a −20 °C freezer, followed by lyophilization for 48 h. Dry samples were removed from the molds and etched with methanol (for 30 min) to induce crystallization and water stability. Following methanol evaporation at room temperature, the scaffolds were then crosslinked in 1-(3-dimetylaminopropyl)-3-etylcarbodiimide hydrochloride (EDC) in N-hydroxysuccinimide (NHS) [20]. Following this, the sponges were further immersed in sodium phosphate dibasic 0.1M Na_2_HPO_4_, neutralized 0.5M NaOH (eliminate the acid environment, which remained from the chitosan solvent) and rinsed several times in deionized water (in preparation for the biological tests). The scaffolds then were frozen again and lyophilized. The vacuum freeze-drying machine ALPHA 1-2 LD plus, MARTIN CHRIST was used (with conditioning −20 °C, 100 Pa, 48 h). Type I collagen solution from bovine skin was prepared by dissolving 0.5% commercially available collagen in water and cross-linked similarly with the EDS solution according to Slovikova et al. [21]. The lyophilized samples were analyzed using several methods.

### 2.2. ATR-FTIR Spectroscopy

A spectrophotometer was used to study the chemical structure, and identify functional groups and their intensity in the obtained scaffolds. The interaction between individual functional groups was evaluated by attenuated total reflection infrared spectroscopy (Genesis II FTIR spectrophotometer (Mattson, Fremont, CA, USA) equipped with an ATR device (MIRacleTM, PIKE Technologies, Fitchburg, WI, USA) with zinc selenide (ZnSe) crystal). All spectra were recorded in absorption mode at 4 cm^−1^ intervals and 64 scans.

### 2.3. Porosity Measurements

Hexane was used as the displacement liquid. The silk fibroin, chitosan sponges and their mixtures with nanohydroxyapatite were immersed in a known volume (*V*_1_) of hexane in a graduated cylinder for 5 min. The total volume of hexane-impregnated sponges along with hexane was recorded as (*V*_2_). The residual hexane volume in the cylinder after the removal of hexane-impregnated scaffolds was recorded as (*V*_3_). The porosity of the scaffolds (ε) was obtained by Equation (1) below [18]:

(1) $$\varepsilon =\frac{\left(V_{1}-V_{3}\right)}{\left(V_{2}-V_{3}\right)}\;.\;$$

### 2.4. Scanning Electron Microscopy

The morphology of lyophilized silk fibroin, chitosan, and nanohydroxyapatite scaffolds with different proportions was studied using scanning electron microscopy (SEM) (LEO Electron Microscopy Ltd., Cambridge, UK). Scaffolds were frozen in liquid nitrogen for 3 min. The software ImageJ 1.33 v W. Rasband NIH USA was used to determine the number and the size of pores. The measurements of all the samples were repeated at different locations.

### 2.5. In Vitro Degradation Test

The in vitro degradation test of scaffolds was performed with 1.5 cm × 0.9 cm samples in 5 mL phosphate-buffered solution (PBS, pH 7.4) at 37 °C containing 1.5 μg/mL lysozyme. The concentration of lysozyme was chosen to correspond to the concentration in human serum [22,23]. Briefly, the lysozyme solution was refreshed daily to ensure continuous enzyme activity and scaffolds were rinsed with distilled water. The degree of in vitro degradation was calculated by the weight loss (2):

(2) $$\mathrm{Weight}\;\mathrm{loss}(\%)=\frac{W_{0}-W_{t}}{W_{0}}\times 100$$

where *W*_0_ is the dry weight before the degradation test and *W_t_* is the dry weight at the time *t*. To degradation dissolution, control samples were stored for 49 days under the same conditions described above, but without the addition of lysozyme.

### 2.6. Mechanical Properties

The resistance to mechanical compression of the nHA/SF/CTS scaffolds has been measured by a Zwick&Roell 0.5 testing machine (Zwick&Roell Group, Ulm, Germany), equipped with a 0.1 kN load cell at room temperature. The crosshead speed was set at 0.5 mm/min. Cylinder-shaped samples 8.7 mm in diameter and 12 mm in height were used. The compressive stress and strain were graphed and the average compressive modulus and standard deviation were determined. The compressive modulus for the scaffold was calculated from the slope of the stress-strain curve at its initial linear section at the strain interval of 1%. The point at which this line crossed the stress-strain curve was defined as the compressive strength of the scaffolds. Measurements were recorded in two different conditions: in a dry state and in a chamber with phosphate-buffered saline (PBS) solution (pH 7.4, thermostated at a temperature of 37 °C).

### 2.7. The Measurements of the Biological Properties

MSCs isolated from bone marrow collected from the iliac plate of a healthy 32-year-old female patient were used in the study by Osyczka, A.M. et al. [24]. Three-dimensional silk fibroin/chitosan matrices with the addition of hydroxyapatite were prepared. The samples were sterilized by UV radiation. MSCs at 10^3^/cm^2^ of biomaterial were seeded on each sample. On the first and fourth day of culture, both 100 μg/mL of potassium 2-ascorbate and 0.1 mM dexamethasone was added to the medium to differentiate the cells into osteoblasts. After 7 days of cultivation, the viability of the cells was determined by the MTS test and the alkaline phosphatase (ALP) was used to determine osteoblastogenesis activity [24].

### 2.8. Statistical Analysis

For each parameter, mean values ± standard error of the mean were calculated (Excel, Microsoft Office 2021, Microsoft, Washington, DC, USA). One-way ANOVA and Kruskal-Wallis were performed for mechanical testing using Past 4.09 software [25]; *p*-values < 0.05 were considered to indicate statistically significant results.

## 3. Results and Discussion

### 3.1. FTIR Spectroscopy

**Figure 1:**
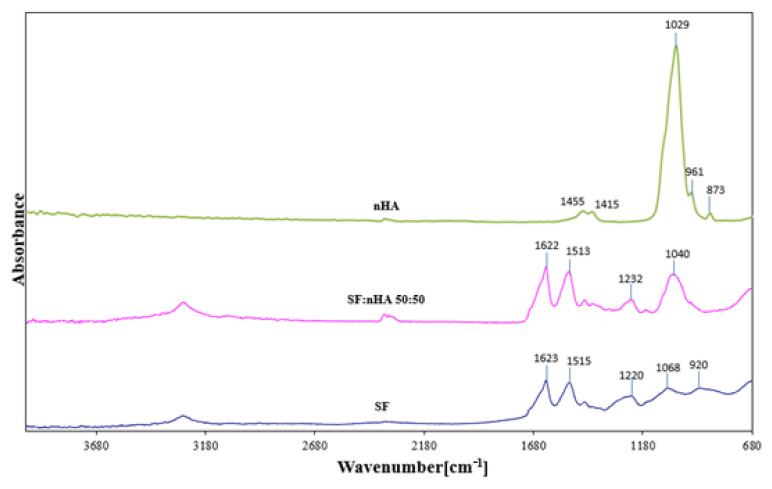
ATR-FTIR spectrogram of silk fibroin (SF), nanohydroxyapatite (nHA), and their blend with weight ratio 50:50.

FTIR spectroscopy is widely used for the study of the structure of polymer materials as well as interactions between polymers. In the spectrogram below (Figure 1) the ATR-FTIR spectra of silk fibroin, hydroxyapatite, and the SF:nHA scaffolds have been shown. The ATR-FTIR spectrum of silk fibroin showed peaks from 3600 to 3100 cm^−1^ which correspond to the –OH stretching and bending vibration mode. In ATR-FTIR spectra of silk fibroin scaffold (after methanol treatment according to Sionkowska A. et al. [22]) the strong absorption bands at 1623 cm^−1^ (amide I), 1515 cm^−1^ (amide II), and 1220–1236 cm^−1^ (amide III) can be observed. The peaks of the nHA, which result from the vibration of the phosphate groups (PO_4_^−^^3^), are also represented at 1029 cm^−^^1^ (*V*_3_ and *V*_1_ mode vibrations of PO_4_^−^^3^), 961 cm^−^^1^ (*V*_3_ and *V*_1_ mode). The band of carbonate (CO_3_^−^, *V*_2_ vibration) can be observed at 873 cm^−^^1^. All bands (summarized in Table 1 and Table 2), are found in the spectra of samples of silk fibroin and hydroxyapatite reported by others [26,27].

**Figure 2:**
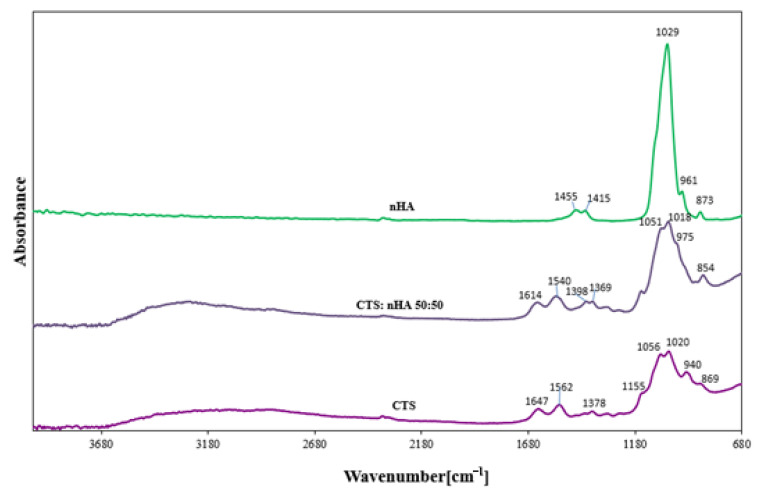
ATR-FTIR spectrogram of chitosan (CTS), nanohydroxyapatite (nHA), and their blend with a weight ratio of 50:50.

In Figure 2 the ATR-FTIR spectra of chitosan and hydroxyapatite and the nHA/CTS scaffolds are shown. The ATR-FTIR spectrum of chitosan showed broad peaks from 2680 to 3680 cm^−1^ which correspond to the –OH stretching and bending vibration mode. In ATR-FTIR spectra of chitosan scaffold, the strong absorption bands at 1647 and 1562 cm^−1^ can be observed, which are attributed to the C = O and -NH_2_ stretching, respectively. The absorption band at 1155 cm^−1^ was assigned to the anti-symmetric stretching of the C-O-C bridge (from β-1,4-glycosidic bond), and is consistent with reports by Ibrahim M. et al. [28].

### 3.2. Porosity

According to Karageorgiou, V. and Kaplan D., porosity and pore size are important determinants of the appropriate properties of future bone formation in vitro and in vivo [29]; therefore, the porosity of the discussed biomaterials was measured.

Table 3 shows the porosity of different nanohydroxyapatite/silk fibroin/chitosan scaffolds. Porosity measurement is crucial for future applications in tissue engineering. High porosity is associated with better connectivity between cells, and improves the diffusion of the active ingredients, and nutrients, the provision of oxygen, removing carbon dioxide from the metabolism, and the creation of space for the growth and proliferation of cells (according to Loh Q.L. and Choong C. [30]). All samples have a high porosity of 60%, starting from the scaffold of pure chitosan, up to the 86% matrix for silk fibroin. All of the porosity values of the blends are above 75% (except for the porosity of chitosan and nanohydroxyapatite samples of about 65%). It can be seen that the addition of an inorganic substance reduces the porosity of the composites while, simultaneously, the addition of silk fibroin leads to increased porosity which, combined with the interconnected porous structure, makes the nanohydroxyapatite/silk fibroin/chitosan scaffolds suitable for tissue engineering.

### 3.3. Scanning Electron Microscopy

**Figure 3:**
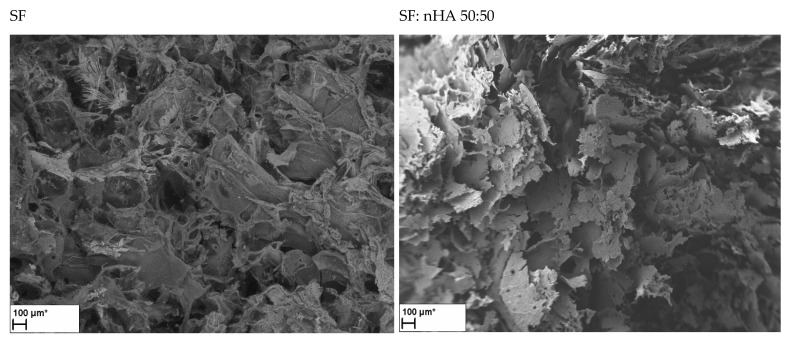

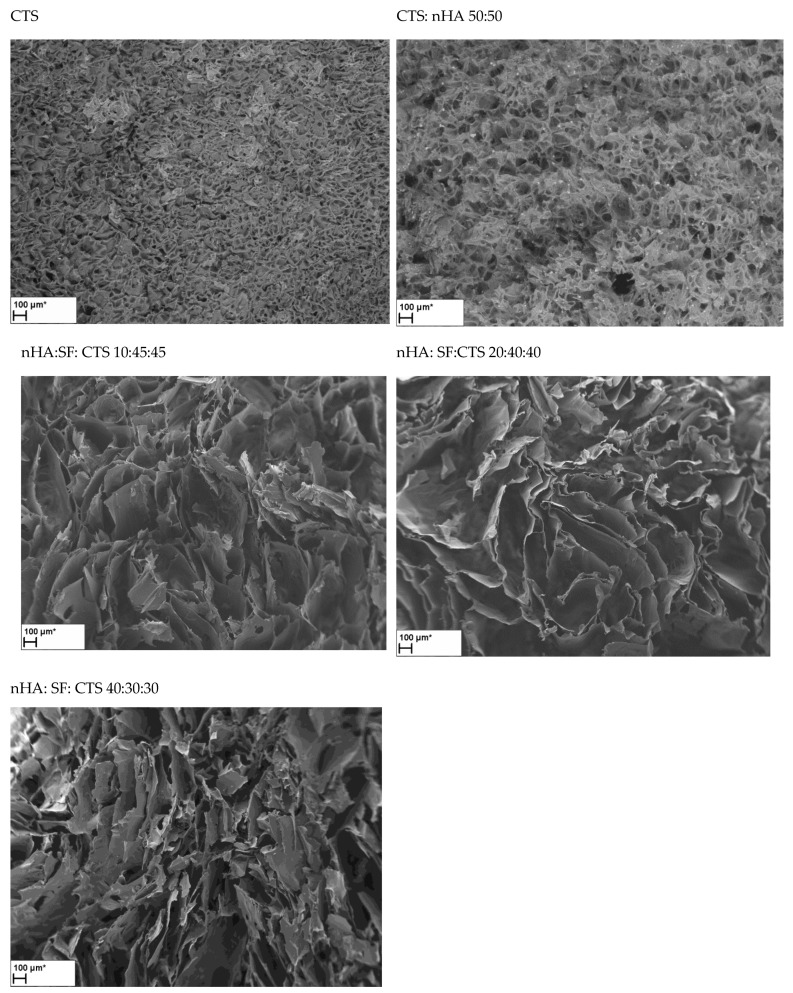
SEM photographs of cross-sections of scaffolds fabricated of single biopolymers and also of their combinations with nanohydroxyapatite (50:50), and ternary matrices of nHA:SF:CTS in the ratios of 10:45:45 and 20:40:40 (scale bar 100 μm).

The size of osteoblasts is on the order of 10–50 μm (Sugawara, Y. et al. [31]); however, osteoblasts prefer larger pores (100–200 μm) for regenerating mineralized bone following implantation (based on results Abbasi, N. et al. [32]). Many scientists (Lim, TC et al. and Murphy, CM et al. [33,34]) reported that a pore size of 100–325 μm was optimal for bone engineering scaffolds in vitro. SEM imaging and ImageJ software were used to characterize the scaffolds. Below are selected SEM images for matrices composed of single biopolymers and their mixtures with nanohydroxyapatite (50:50) and ternary matrices of nHA:SF:CTS in the ratios of 10:45:45; and 20:40:40 are presented (Figure 3). We have published the SEM pictures for matrices with other proportions previously [17,22]. The pore size, shape, surface, and wall thickness are summarized in Table 4.

The matrix, fabricated entirely of chitosan, was characterized by the smallest pores of 80 µm with a rounded shape. However, for mixtures with nanohydroxyapatite, silk fibroin, and chitosan, we can observe larger pore sizes, and both their shapes and sizes vary. In addition to the rounded holes, we can see spindle-shaped pores, and elongated holes with parallel walls positioned closely to each other. We can see different pore sizes, from 100 to 300 and also 500–600 µm for one type of matrix (it can be most clearly observed for the proportions nHA:SF:CTS 40:30:30 and 15:70:15). The presence of both small and large pores has positive aspects for bone regeneration. This was noted by Di Luca et al. [35] who created gradients in three-dimensional scaffolds. According to their research, the scaffolds with a gradient in the porosity of the poly ε-caprolactone (PCL) scaffolds improved the osteogenic differentiation of human mesenchymal stem cells (MSCs) in vitro by increasing the calcium content and ALP activity because of the better supply of oxygen and nutrients in larger pores.

### 3.4. Degradation Properties

It is important to understand the degradation profile of a scaffold under conditions simulating human bodily fluids because the degradation time of the matrix must be consistent with the process of new bone formation. The idea behind the biopolymer is that it will serve as a scaffold at the start of the regeneration process, and then to give way to the newly-formed bone. The broken bone regeneration process can be divided into several stages. During the first seven days (for small bone defects), there is an increase in elements such as zinc and magnesium at the fracture site. After 14 days, it is possible to observe the formation of collagen fibers, and 21–28 days following mineralization breakage of the fibers, slow bone reconstruction, and the formation of solid trabeculae [36].

**Figure 4:**
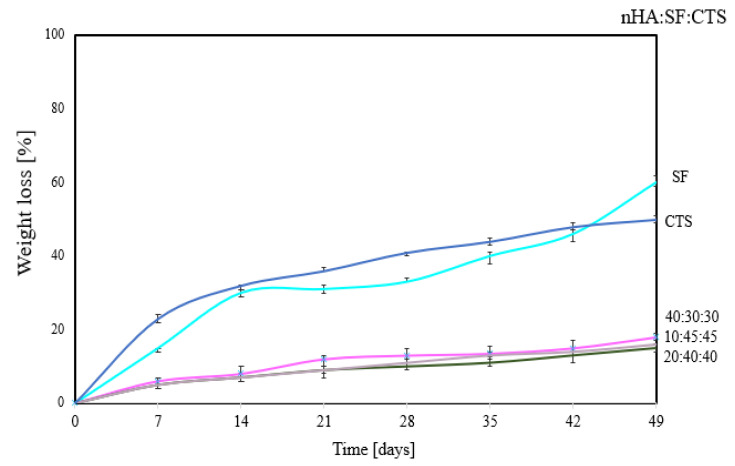
Weight loss [%] of nHA/SF/CTS scaffolds with different weight ratios (*w*/*w*) of components (10:45:45; 20:40:40; 40:30:30) in lysozyme solution (1.5 μg/mL).

The weight loss of scaffolds in lysozyme solution (during seven weeks of measurements) is shown in Figure 4.

It can be seen that the 100% single biopolymer matrix undergoes degradation at the highest rate. After 7 weeks, the matrix of silk fibroin degrades by 60% and that of chitosan by 50% in lysozyme solution. The most stable is the matrix with the proportion nHA:SF:CTS 20:40:40; 10:45:45; 40:30:30, which lost approximately 10% of its mass after 7 weeks. This is a different tendency than for measurements with the use of nHA:SF:CTS scaffolds in the 70:15:15; 15:70:15; 15:15:70 ratio presented in the literature [12]. Where the samples 70:15:15 and 15:70:15 degraded significantly (after 21 days), only sample 15:15:70 remained stable and did not degrade (having similar properties as samples in Figure 4). It proves a better correlation, and stronger interactions between the components of nHA:SF:CTS in the mixture with the ratio 10:45:45; 20:40:40; 40:30:30. However, a mere 10% of weight loss after seven weeks of treatment of the matrices with lysozyme solution is not a sufficient level considering the nature of new bone formation.

### 3.5. Mechanical Properties

**Figure 5:**
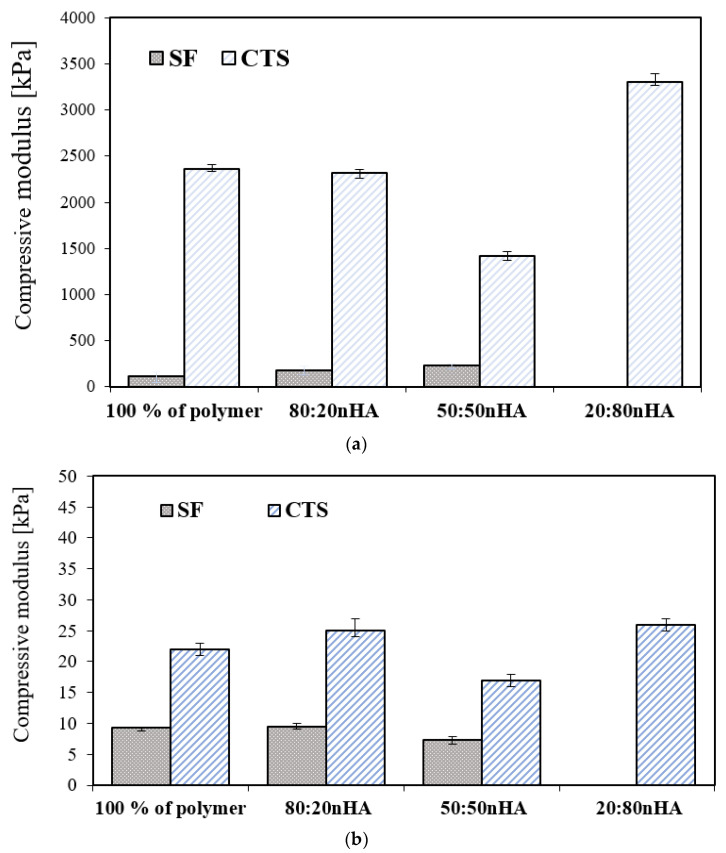
(**a**) Compressive modulus of nHA and polymers with different weight ratios (*w*/*w*) (80:20; 50:50; 20:80) in a dry state; *p* < 0.05. (**b**) Compressive modulus of nHA and polymers with different weight ratios (*w*/*w*) (80:20; 50:50; 20:80) in solution of PBS, pH 7.4, temp. 37 °C; *p* < 0.05; *p* < 0.05.

**Figure 6:**
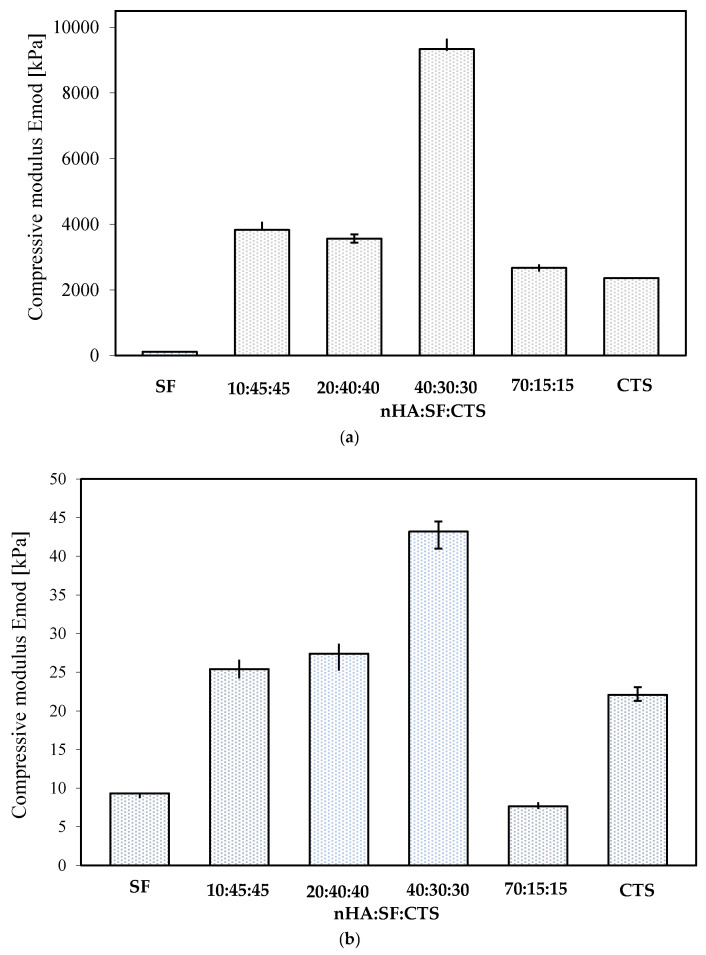
(**a**) Compressive modulus of nHA/SF/CTS scaffold with different weight ratios (*w*/*w*) (10:45:45; 20:40:40; 40:30:30; 70:15:15) in a dry state; *p* < 0.05. (**b**) Compressive modulus of nHA/SF/CTS scaffold with different weight ratios (*w*/*w*) (10:45:45; 20:40:40; 40:30:30; 70:15:15) in solution of PBS, pH 7.4, temp. 37 °C; *p* < 0.05.

The mechanical properties of scaffolds with all compositions were tested under compression under dry conditions (Figure 5a and Figure 6a) and in the solution of PBS, pH 7.4, temp. 37 °C (Figure 5b and Figure 6b). It can be seen that the most resilient to crushing was the chitosan sample with the addition of a mineral component. On the contrary, the compressive modulus for the scaffold decreases with silk fibroin. The values of mechanical parameters measured in the solution of PBS condition (Figure 5b and Figure 6b) are far lower than those presented under dry conditions. The compressive modulus significantly decreases. The results of the measurements in the solution phase showed that the difference between the compressive modulus of the silk fibroin and chitosan samples is not as significant, compared to the properties of dry samples. The sample with the proportion of 40:30:30 (nHA:SF:CTS) shows the highest resistance to compression. Nevertheless, all of the samples (except SF:nHA 20:80) are still capable of withstanding the implantation process and can also act perfectly to adjust to the desired cavity.

### 3.6. Results of the Measurements of Biological Properties

**Figure 7:**
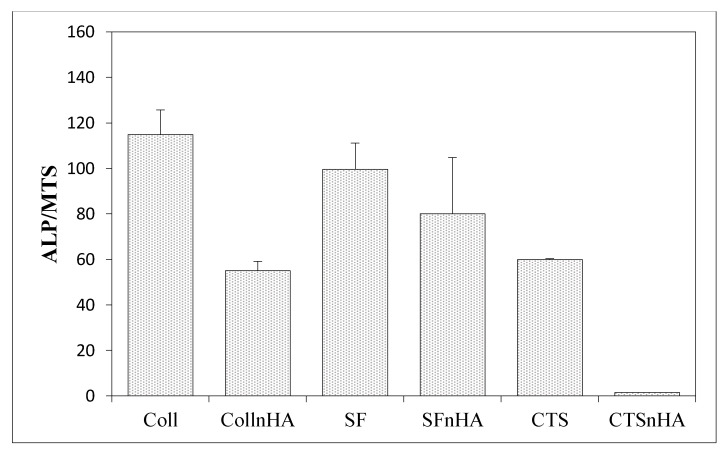
Results of in vitro studies on human mesenchymal stem cells using silk fibroin and chitosan materials; ALP activity per cell.

**Figure 8:**
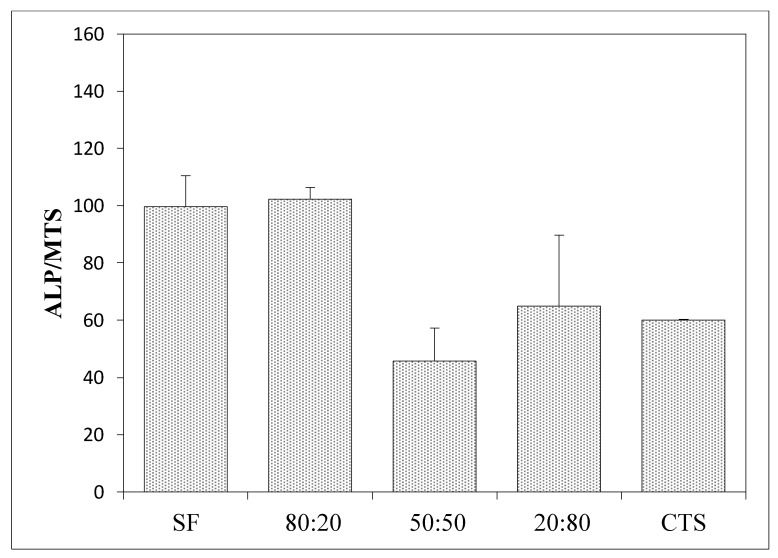
Measurement of the alkaline phosphatase activity concerning cell viability/(MTS) on biomaterials for seven days in the presence of an osteogenesis-stimulating medium.

**Figure 9:**
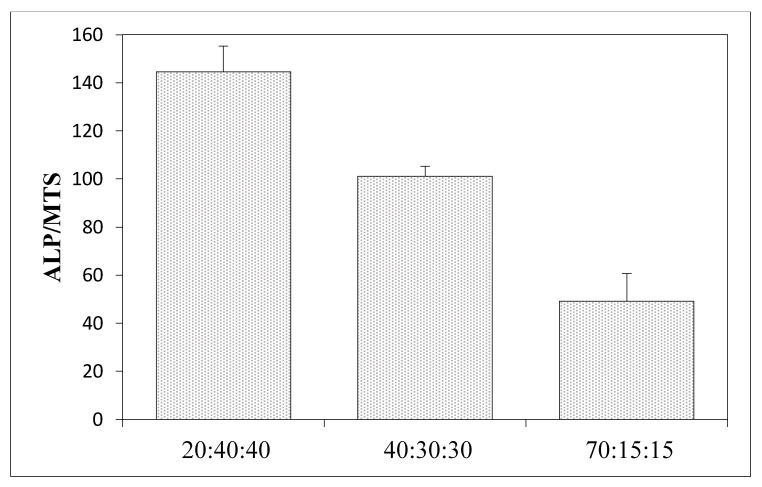
Results of in vitro studies on human mesenchymal stem cells using materials of hydroxyapatite, silk fibroin, and chitosan; ALP activity per cell.

The biological properties using human mesenchymal stem cells (MSCs) were measured to determine how a given material affects the level of cell differentiation, especially toward in the case of osteogenic cells (Figure 7, Figure 8 and Figure 9).

Figure 7 and Figure 9 show the results of the alkaline phosphatase (ALP) activity per cell depending on the variable amount of hydroxyapatite in the composite. Figure 7 shows the ALP phosphatase activity values for composites made of 100% collagen, silk fibroin, and chitosan, and how this value changes following the addition of hydroxyapatite. In all cases, the addition of hydroxyapatite reduces the ALP activity by more than 60% compared to scaffolds without hydroxyapatite. Figure 9 concerns three-component scaffolds (nHA:SF:CTS), in which the trend of decreasing ALP activity with the addition of hydroxyapatite is also noticeable. The scaffold with 20% of hydroxyapatite content showed ALP activity (over 100 units), while the composite with 70% of hydroxyapatite content demonstrated half of this ALP activity (around 50). However, it is still greater than for the two-component scaffolds (SF:nHA and CTS:nHA 50:50).

**Figure 10:**
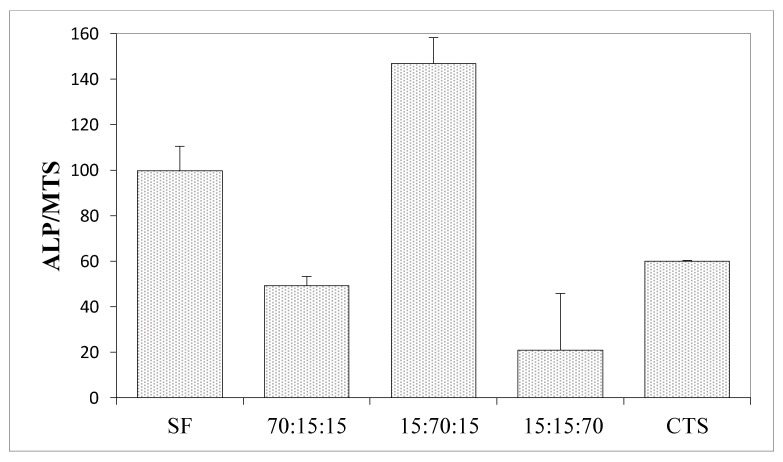
Results of in vitro studies on human mesenchymal stem cells on materials of hydroxyapatite, silk fibroin, and chitosan; ALP activity per cell.

ALP is an early marker of the process of osteogenic cell differentiation and its increased level accompanies the early stages of differentiation of the osteogenic cell phenotype. The highest activity of alkaline phosphatase (ALP) per cell was recorded for the nHa:SF:CTS 15:70:15 scaffold (around 150) (Figure 10), which is a higher value than for the pure collagen samples or collagen mixtures with nanohydroxyapatite. Equally, high ALP values were shown by the samples from pure silk fibroin and the mixture of SF:CTS 80:20. It can be seen that these matrices are similar in nature (rough), shape (oblong, spindle-shaped), and pore size (100–300 μm and 500–600 μm). Moreover, similar properties are shown by a composite sample with a weight ratio of 20:40:40 whose ALP/MTS values are also very high.

Higher values of alkaline phosphatase (ALP) per cell for scaffolds containing higher amounts of silk fibroin may indicate a good biological potential of this protein, higher than for the collagen or chitosan scaffolds tested in the above. This can be explained by the fact that the pH value of the silk fibroin solution (obtained in an alkaline medium) favors the activity of alkaline phosphatase. Silk fibroin fabrics are highly porous, have a large surface area, and absorb more water. Additionally, focusing on the mechanism for the formation of crystallinity centers, during bone mineralization, the distance between individual crystallinity centers is given as 0.362 nm according to Pawlikowski [36], which is consistent with the distances between amino acids alternately arranged in the beta-sheet structures, in silk fibroin according to Sashina [37]. Additionally, the amino acid composition of silk fibroin rich in sericin is similar to the composition of growth factors, commonly added to increase the biocompatibility of materials in tissue engineering [38].

The shape and size of the pores also draw attention to materials with the highest ALP/MTS value. Elongated, spindle-shaped pore shapes are crucial for bone reconstruction applications. This issue has been investigated in the literature in relation to other biomaterials [39,40,41].

To sum up, the addition of silk fibroin displays huge potential regarding bone reconstruction engineering. Its similar structure to collagen makes it a potential replacement and shows excellent promise for orthopedic tissue engineering.

## 4. Conclusions

Three-dimensional scaffolds based on the blend of nanohydroxyapatite, silk fibroin, and chitosan can be successfully prepared by using the lyophilization technique and has the potential to become an alternative material to bovine type I collagen scaffolds. The degradation time studies showed that the addition of chitosan prolongs the sample disintegration. In vitro studies with human mesenchymal stem cells have proven the high level of biotolerance of the produced materials, especially for the 20:40:40 and 15:70:15 (nHa:SF: CTS) ratios. The presence of silk fibroin and elongated, spindle-shaped pore shapes positively influenced the differentiation of cells into osteogenic cells, whereas nHA inhibited the cell differentiation-the more nHA in the material, the lower the ALP/MTS value. The highest activity of alkaline phosphatase per cell was recorded for the nHa:SF:CTS 15:70:15 scaffold.

It cannot be stated unequivocally that a particular component either increases or reduces the activity of ALP. Only the right combination of all three ingredients and in-depth research of the newly-fabricated composite may provide full information about the biological properties of the material.

## Figures and Tables

**Table 1:** The position of characteristic bands in FTIR-ATR spectra of silk and nanohydroxyapatite, and their composites, according to [27].

Assignments	Observed Vibrational Frequencies Wavenumber [cm^−1^]		
	SF	SF:nHA 50:50	nHA
H_2_O absorber., N-H stretching (A amide)	3285	3282	
C-H (B amide)	3095	-	
C = O stretching (I amide) intermolecular β structure	1623	1622	
60% N-H bending; 40% C-N stretching (II amide) (after Methanol treatment)	1515	1516	
CH_2_ scissioring	1447	1446	1455
CH_2;_ COO^−^	1411	1408	1415
CH_3_ wagging; O-H bending (from Serine)	1340	1335	
30%C-N< stretching; 30% N-H bending, 10% C = O stretching; 10% O = C-N bending; 20% other (III amide)	1220–1236	1232	
-C-O-C stretching asymm.	1168	1167	
PO_4_^3−^	-	1040	1029
HPO_4_^2−^	-	-	961
HPO_4_^2−^/CO_3_^−^	-	958	873

**Table 2:** The position of characteristic bands in FTIR-ATR spectra of chitosan and nanohydroxyapatite, and their composites, according to [27].

Assignments	Observed Vibrational Frequencies Wavenumber [cm^−1^]		
	CTS	CTS:nHA 50:50	nHA
H_2_O absorber., N-H (A amide)	3320	3296	
C-H (B amide)	3085	-	
C-H stretching	2886	2873	
C = O stretching (I amide)	1647	1636	
60% N-H bending; 40% C-N stretching (II amide)	1562	1540	
>CH_2_ scissioring	-	-	1455
>CH_2_	1411	1407	1415
CH_3_ in amide group	1378	1369; 1398	
-C-O-C stretching asymm.	1155	1167	
=CO stretching skeletal vibration	1056; 1020	1051	
PO_4_^3−^	-	1018	1029
HPO_4_^2−^	-	975	961
HPO_4_^2^^−^/CO_3_^2^^−^	-	854	873

**Table 3:** The porosity of silk fibroin and chitosan scaffolds and their complex with nanohydroxyapatite.

	Porosity [%]
SF	86
SF:nHA 50:50	75
CTS:nHA 50:50	65
CTS	70
nHA/SF/CTS 10:45:45	85
nHA/SF/CTS 20:40:40	80
nHA/SF/CTS 40:30:30	75

**Table 4:** Characterization of materials fabricated in this research.

nHA:SF: CTSRatio	Average Pore Size [μm]	Shape	Wall Thickness [μm]	Surface (Smooth/Rough)
SF	140–220	Multi-shaped and round	0.3–1.5	very rough and extensive surface
SF:nHA	110–200	Multi-shaped and round	1	very rough
CTS	40–80	round	1–3	smooth
CTS:nHA	80	Multi-shaped and round	2–5	rough
SF: CTS 80:20	50–160 with a predominance of 90	Multi-shaped	3–5	rough
SF: CTS 50:50	60–160	Multi-shaped	1–5	smooth and rough in places
SF: CTS 20:80	150 and 325	Oblong and round	1–5	smooth
nHA:SF:CTS 20:40:40	175–375	Oblong, spindle-shaped	0.5–3	smooth
nHA:SF:CTS 40:30:30	125–225 and also 600	Oblong, spindle-shaped, narrow, parallel to each other, and round	1–4	smooth
nHA:SF:CTS 70:15:15	110	round	Mostly 2, but there are also 7	smooth
nHA:SF:CTS 15:70:15	100–300 and 500–600	Oblong, spindle-shaped	Mostly 5, but there are also 2, 10	rough
nHA:SF:CTS 15:15:70	175	round	Mostly 2, but there are also bigger 10–13	smooth and rough in places

## Data Availability

The data presented in this study are available on request from the corresponding author. Data can be available under the request.
